# Tunable analog thermal material

**DOI:** 10.1038/s41467-020-19909-0

**Published:** 2020-11-27

**Authors:** Guoqiang Xu, Kaichen Dong, Ying Li, Huagen Li, Kaipeng Liu, Longqiu Li, Junqiao Wu, Cheng-Wei Qiu

**Affiliations:** 1 Department of Electrical and Computer Engineering, National University of Singapore, Kent Ridge, 117583 Republic of Singapore; 2 Department of Materials Science and Engineering, University of California, Berkeley, Berkeley, CA 94720 USA; 3 Materials Sciences Division, Lawrence Berkeley National Laboratory, Berkeley, CA 94720 USA; 4 Interdisciplinary Center for Quantum Information, State Key Laboratory of Modern Optical Instrumentation, College of Information Science and Electronic Engineering, Zhejiang University, Hangzhou, 310027 China; 5 ZJU-Hangzhou Global Science and Technology Innovation Center, Key Lab. of Advanced Micro/Nano Electronic Devices & Smart Systems of Zhejiang, Zhejiang University, Hangzhou, 310027 China; 6 State Key Laboratory of Robotics and System, Harbin Institute of Technology, Harbin, 150001 China

**Keywords:** Metamaterials, Mechanical properties, Applied physics

## Abstract

Naturally-occurring thermal materials usually possess specific thermal conductivity (*κ*), forming a digital set of *κ* values. Emerging thermal metamaterials have been deployed to realize effective thermal conductivities unattainable in natural materials. However, the effective thermal conductivities of such mixing-based thermal metamaterials are still in digital fashion, i.e., the effective conductivity remains discrete and static. Here, we report an analog thermal material whose effective conductivity can be in-situ tuned from near-zero to near-infinity *κ*. The proof-of-concept scheme consists of a spinning core made of uncured polydimethylsiloxane (PDMS) and fixed bilayer rings made of silicone grease and steel. Thanks to the spinning PDMS and its induced convective effects, we can mold the heat flow robustly with continuously changing and anisotropic *κ*. Our work enables a single functional thermal material to meet the challenging demands of flexible thermal manipulation. It also provides platforms to investigate heat transfer in systems with moving components.

## Abstract (web summary)

Thermal metamaterials are able to produce unconventional physical properties. Here, the authors demonstrate a thermal metamaterial with conductivity that can be continuously tuned over a very large range.

## Introduction

**Fig. 1:**
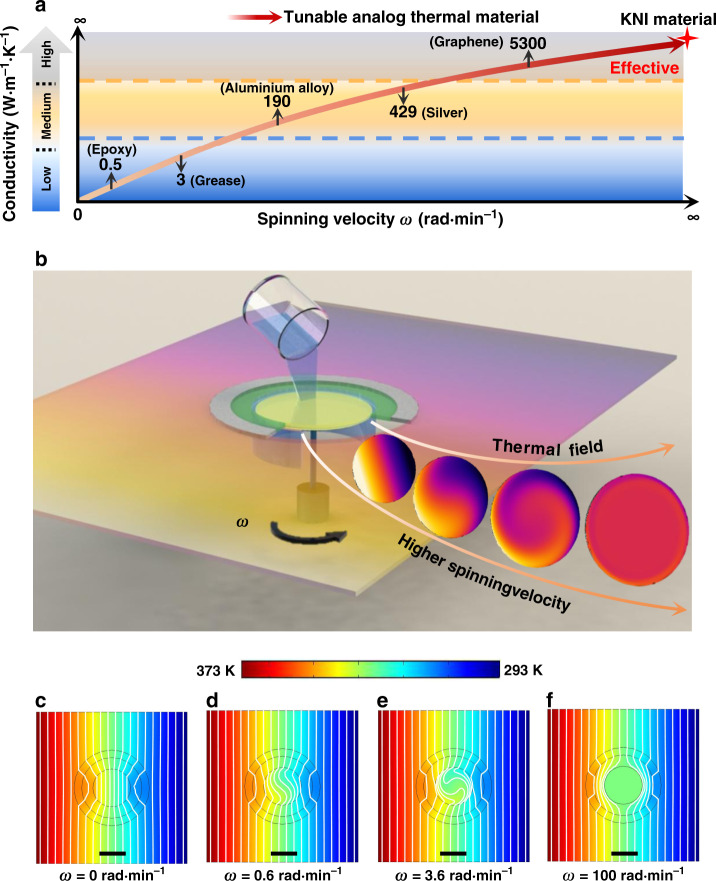
Principle of tunable analog thermal material and representative modulated cases. (**a**) The current progress of thermal material, while KNI denotes the material with infinite thermal conductivity (*κ*). The background colors indicate the changing trends of conductivities. It indicates that each discrete conductivity only corresponds to one conventional thermal material; (**b**) the schematic of tunable analog of thermal material induced by spinning fluid. The employed fluid is infused into the central region of the entire system and actuated by a spinning disk (marked by yellow) under the fluid surface. In such operations, tunable finite and effectively infinite conductivities can be observed with corresponding rotational excitations. (**c**)–(**f**) respectively illustrate the thermal profiles of the modulated schemes 1~4 under specific rotation rates. The scale bar is 40 mm (black lines). Among them, (**c**) is the enhanced transparency at *ω* = 0 rad∙min^−1^ (scheme 1); (**d**) denotes the field contortion at *ω* = 0.6 rad∙min^−1^ (scheme 2); (**e**) presents the inverse field distribution at *ω* = 3.6 rad∙min^−1^ (scheme 3); and (**f**) exhibits the sensitive cloaking with effectively large conductivity at *ω* = 100 rad∙min^−1^ (scheme 4).

Various kinds of metamaterials are emerging to meet the increasing demands on field manipulation. As a major diffusive member, thermal metamaterial reveals enormous potential on motivating the current thermal techniques, while the pioneering electromagnetic metamaterial of field invisibility^1,2^ inspires the thermal analogs of thermal cloak^3–11^, concentration^12–15^, rotation^16,17^, (enhanced) transparency^18–20^, and illusion^21–23^. Static thermal metamaterials are mostly designed with natural materials, whose inherent conductivities only take some discrete digital values as illustrated in Fig. 1a. For certain functionality, some new digital conductivities and specific inhomogeneities can be effectively realized through mixing natural thermal materials based on the transformation methods^1,3–6,13–15,17,18,21,22,24^ or scattering cancellation method^2,7–10,12,16,18–20^. Based on the conventional design principle, these new digital materials still possess non-tunable thermal conductivities and fixed anisotropies, which bring great challenges in the adjustment and functional switching of thermal manipulations. Though the propositions of macroscopic thermal diodes^24^, encoded^25^, and doublet^26^ schemes can be alternative solutions of switching conductivities, the complicated fabrications and the narrow tunable range limited by Maxwell-Garnett mixing rule are the major restrictions in practice. Recently, a class of thermal material with effectively infinite conductivity (*κ*-near-infinity, KNI) is proposed^27^. KNI breaks the conductive limitation of conventional (meta)material by introducing extreme convection. However, it is only valid to attempt the most upper-right regime of Fig. 1a. Furthermore, the extreme convection would suppress the possibility of inhomogeneity in the effective thermal conductivity profile, thus eliminating the advanced control of heat flow with inhomogeneous thermal conductivities.

Considering the conventional thermal materials and KNI material shown in Fig. 1a, one would be curious whether the entire range of conductivities can be strung within a single functional material to fill the gap of continuous tunability in conductive components, which is like an analog signal in electronics. Here, we propose that a spinning medium within one system could be an efficient solution. Spinning components are known to be able to greatly modify the behaviors of physical fields, as the successful demonstrations of anti–parity–time symmetry heat transfer in thermodynamics^28,29^, and nanoparticle sensing^30^, non-reciprocal light propagations^31–34^, topological insulator^35–37^ in photonics and acoustics. It is expected that in analogy, the extra freedom of rotation rates could also be employed in thermal materials to create the desired tunability.

In this paper, we propose the tunable analog thermal material by spinning uncured PDMS in a bilayer structure. The jelly-like medium of PDMS could hold the fluid, which plays the convective role, firmly within it when mechanical rotating-speed is moderate, thus significantly avoiding the uneven flows. We theoretically provide the relations between integrated spinning fluid and local effective conductivities/field deflections of such system. Active thermal manipulations under varied conductive demands, including enhanced transparency, field contortion, inverse field distribution, and sensitive cloaking, are representatively confirmed, showing continuous tunability ranging from near-zero to near-infinity and high conveniences that are lacking in conventionally static thermal (meta)materials. The findings fill the gap of achieving active tunable conductivities/anisotropies and functional switching only by manipulating a homogeneous material layer.

## Results

### Principle of tunable analog thermal material

In the previous cloaking researches^3–11^, the aim is to create a cloaking region without temperature gradients. Thus leading to the preferential cloaking regions with highly homogeneous conductivity (such as steel, silver, etc.) both in the transformation-dependent, and scattering and cancellation based designs. Such operations are the favorite means of eliminating the effects of inlet thermal energy and assisting the functional cloaking layers. Similar to the thermal cloaking, small (near-zero) conductive region (such as epoxy, grease, etc.) is the prior choice to achieve approximate/enhanced temperature gradients in the design of (enhanced) transparency^18–20^ (concentration^12–15^). These behaviors of no or enhanced (approximate) temperature gradients can be observed with single homogeneous and finitely conductive materials. However, the homogeneous and finite conductivities restrict the potential of thermal materials, as simple effects without further field manipulation cannot satisfy varied demands in latent applications. The imperfection of homogeneous thermal materials contributes to the improvement of inhomogeneous metamaterials. Considering specific spatial transformations, the pre-designed field contortions can be demonstrated with anisotropic conductivities. Moreover, KNI materials further provide the possibility of creating effectively infinite conductivities to break the limitations of conventional thermal materials. The current thermal materials only indicate the independent, irreversible, and non-adjustable progress of low or high, homogeneous or inhomogeneous, and finite or infinite conductivities in specific cases. It seems difficult to simultaneously and adjustably satisfy these inconsistent conductivities within a single material layer in one system.

The seemingly contradictory conductivities can be easily excited through spinning a single fluid. A static fluid with an inherent (near-zero) conductivity can gather temperature gradients from outside. On the other hand, an effectively large conductivity can be also reached by imposing extreme convection in a fluid domain, though the effective inhomogeneity is suppressed by the rapid homogenization of temperature fields^27^. It is straightforward to anticipate that modulated conductive profiles could be also effectively obtained when the fluidic rotation slows down. However, some uncontrollable effects like viscosity and inhomogeneous flow would cause the modulation of conductivities and thermal profiles to fail at moderate rotation rates with the KNI strategy^27^. For example, the rotating flow actually leads to the inhomogeneous distribution of fluid flow, which significantly contributes to the non-zero fluid temperature gradients and field deflections at the boundaries of the fluid and its surroundings at a moderate velocity. Thus, the thermal cloaking would fail with external field perturbations at a moderate velocity^27^ or be switched to non-cloaking behaviors with non-zero temperature gradients in the target region (see Supplementary Fig. 3). The problem is how to suppress these unwanted effects and simultaneously make the analog conductivity inhomogeneous and tunable in-situ. Inspired by the water-holding effect of jelly-like medium, these inconveniences can be readily solved, since it firmly holds fluid within as the convective role without the issue of uneven flows. Hence, a jelly-like fluid with a small conductivity is an ideal objective for creating tunable analog thermal material at arbitrary rotation rates.

To highlight the thermal behaviors of tunable analog thermal material, bilayer structure^7^ is employed to cancel the external field distortion. To eliminate external field distortion, a rigorous analysis is implemented for deriving the matching conditions between adjacent regions based on thermal conduction and convection equations (see Supplementary Notes 1–3). As a specific case of thermal manipulation with spinning fluid at extremely large rotation rate, KNI material^27^ is proposed to deal with the finite thermal conductivity and highly conductive background. Combining with the conventional bilayer structure and fluid mobility, tunable conductivities can be extensively modulated under a general rotation rate, thus contributing to the non-distortion field and highlighting the tunable performances in a simple system. The entire spinning system shown in Fig. 1b, consisting of background, bilayer structure, and central fluid region (excited by a spinning disk), is proceeded under uniform heat flux density generated by the constant high and low temperature at two opposite boundaries.

The entire spinning system shown in Fig. 1b, consisting of background, bilayer structure, and central fluid region (excited by a spinning disk), is proceeded under uniform heat flux density generated by the constant high and low temperature at two opposite boundaries. The problem here is how to satisfy a specific conductive demand with a certain rotation rate. Different from KNI material, the moderate angular velocity is essential for simultaneously keeping finite conductivity and contorting temperature field distribution. Hence, field deflection and rotation caused by spinning effects and thermal convection should be considered. That is, the effective conductivity and heat flux deflection at the interface between the spinning fluid and static solid domains are the keys. Since the interior objective is fluid, the analysis can be operated with the thermal convection equation. Besides, the velocity along radial direction is ignored, as only rotation is employed in the target region. For simplification, an independent angular velocity (*ω*) is considered, and thus the governing function^38^ in the central fluid domain can be expressed as: $\frac{{\kappa _0}}{{\rho _0c_0}}\left( {\frac{{\partial ^2T}}{{\partial r^2}} + \frac{{\partial T}}{{r\partial r}} + \frac{{\partial ^2T}}{{r^2\partial \theta ^2}}} \right) = \omega \frac{{\partial T}}{{\partial \theta }}$, where *ρ*_0_, *c*_0_, and *κ*_0_ respectively denote the density, specific heat, and inherent thermal conductivity of the employed fluid*, r* and *θ* are the radial and azimuthal locations, and *ω* is the angular velocity of the fluid region. Note that ∂*T*/∂*θ* at the interface of the spinning fluid domain and the internal layer (of the bilayer structure) approaches to zero once a large angular velocity is excited^27^. Thus, its effect is not obvious for the sensitive cloaking behaviors with few temperature gradients. However, this component should be involved in the manipulative cases with finite rotation rate, i.e., finite effective conductivity. Considering the effectively inhomogeneous temperature distribution and field continuity, the local effective conductivities along the principle axes at the interface can be written as (see Supplementary Note 2):

1 $$\kappa _{0,r}^{eff} = R_0\sqrt {\omega \rho _0c_0\kappa _0} \cdot \frac{{\cos \left( {\theta - \phi (\omega ,r) + \frac{\pi }{4}} \right)}}{{\cos \left( {\phi (\omega ,r)} \right)\cos \left( \theta \right)}}.\quad \left( {\omega \,\ne\, 0} \right)$$

2 $$\kappa _{0,\theta }^{eff} = 2R_0\sqrt {\omega \rho _0c_0\kappa _0} \cdot \frac{{\sin \left( {\theta - \phi (\omega ,r) + \frac{\pi }{4}} \right)}}{{\cos \left( {\phi (\omega ,r)} \right)\sin \left( \theta \right)}}.\quad \left( {\omega\, \ne \,0} \right)$$

In Eqs. (1) and (2), *R*_0_ is the radius of the fluidic region, while *ϕ*(*ω, r*) is a continuous real function of *ω* and *r* which can be defined in the general solution and calculated in the convective process. Such tunable conductivities can be also used to describe the actual fluid domain through taking the corresponding radial components. The local effective conductivities are the functions of system azimuths (depend on the inlet heat flux) and angular velocity. Regarding the underlying field deflection, the relation of $\phi \left( {\omega ,r} \right) = \phi \left( {\sqrt {\frac{{\omega \rho _0c_0}}{{\kappa _0}}} r} \right) = {\mathrm{arctan}}\left( {bei_1\left( {\sqrt {\frac{{\omega \rho _0c_0}}{{\kappa _0}}} r} \right)/ber_1\left( {\sqrt {\frac{{\omega \rho _0c_0}}{{\kappa _0}}} r} \right)} \right)$ should be employed at a finite angular velocity based on Kelvin’s function^39^. Once the rotation rate is large enough, the value of *ϕ*(*ω*, *r*) approaches π/4 to maintain the approximate deflections of the spinning fluid domain and the internal layer^27,38^. Note that, Eqs. (1) and (2) are available when convection exists, i.e., *ω* ≠ 0. Besides, Eqs. (1) and (2) indicate that effectively conductive distributions are anisotropic at a general angular velocity. The reason for this is the non-negligible effect induced by the fluidic rotation with a general-speed convection. Different from the KNI material with extreme convections^27^, the cases excited by generally convective velocities might not possess sufficient field couplings to realize the rapid homogenization of thermal profiles with few temperature gradients. Thus, the anisotropic field distributions with non-zero temperature gradients are the major behaviors of field manipulations at this stage. Besides, these effectively directional conductivities further provide the evident explanations of rotating field distributions shown in Fig. 1d,e, and the effectively near-infinite conductivity of KNI material^27^ can be regarded as their approximation without temperature gradients, only when extreme convections are employed (see Supplementary Note 2). In general, such anisotropically effective thermal conductivities provide a significant tool for achieving various functions of field manipulations at arbitrary angular velocities.

For creating tunable conductivities with spinning fluid, the temperature field deformation caused by heat flux deflection^40–42^ at the liquid-bilayer interface is the dominant term. Such effective heat flux deflection can be obtained with the effective conductivity of the fluid domain and expressed as follows (see Supplementary Note 3):

3 $$\theta _{{\mathrm{bend}}} = \arctan \left( {\frac{{q_y^\theta }}{{q_x^\theta }}} \right) \\ = \arctan \left( {\frac{{\left( { - \kappa _{0,xx}^{eff}\left( {\theta ,\omega } \right) + \kappa _{0,yx}^{eff}\left( {\theta ,\omega } \right)} \right)\cos \theta \sin \theta }}{{\kappa _{0,xx}^{eff}\left( {\theta ,\omega } \right) \cdot \cos ^2\theta + \kappa _{0,yx}^{eff}\left( {\theta ,\omega } \right) \cdot \sin ^2\theta }}} \right).$$

Both the effective conductivity and heat flux deflection are the functions of angular velocity and azimuthal component, once the spinning fluid is determined. Owing to the fluid continuity and mobility, thermal energy at the liquid-bilayer interface would be rotationally taken into the center under spinning effects, which contributes to the programmable thermal conductivities at certain rotational rates.

### Numerical demonstration of tunable analog thermal material

To demonstrate such tunable conductivities excited by spinning fluid, a bilayer structure is proposed and fabricated. The simulated temperature distributions at different angular velocities are shown in Fig. 1c–f. At the beginning, the case of enhanced thermal transparency (scheme 1) is first exhibited without spinning effect, i.e., *ω* = 0 rad min^−1^. As presented in Fig. 1c, the near-zero conductivity *κ*_0_ inside the center contributes to the behaviors of enhanced transparency. It indicates that significantly uniform temperature gradients are observed in the central region, and no field distortions occur in the background. Furthermore, other manipulations are also respectively demonstrated with increasing rotation rates as shown in Fig. 1d, e. Note that turbulent effects are ignored since the angular velocities are small enough in these cases (*ω* = 0.6 rad min^−1^ and *ω* = 3.6 rad min^−1^). The case of field contortion (scheme 2) shown in Fig. 1d is excited when the rotation rate is 0.6 rad min^−1^. Different from the rotated behaviors in static homogeneous solids, the deflection here behaves as ‘wave’ distributions due to the fluid continuity and mobility. Further increasing the angular velocity, the field deflection would concomitantly increase. The internal thermal field presented in Fig. 1e is inversed to the background temperature distribution by the spinning fluid when the angular velocity approaches 3.6 rad min^−1^. That is, the hot and cold locations in the center are corresponding to the low and high temperature boundaries of the entire system, and the propagated direction of temperature gradient inside the center is inverse to the external background. Hence, the case of exhibiting inverse thermal distribution (scheme 3) can be obtained in the spinning fluid domain without additional sources. Further accelerating the fluid rotation, the behavior of sensitive cloaking (scheme 4) can be also observed with effectively large conductivities (*ω* = 100 rad min^−1^) as shown in Fig. 1f. Owing to the temperature homogenizations by the spinning fluid, the fluid domain showcases a uniform thermal profile with few temperature gradients. It is worthy to note that such sensitive cloaking behaviors can be widely observed in the schemes with sufficiently large angular velocities. At these stages, the effectively directional conductivities are far larger than those of the background and bilayer structure. Thus, few or no temperature gradients exist due to these effectively large conductive components, and the temperature fields inside the fluidic region can be rapidly homogenized by the high convective effects. Thus, it can be anticipated that the KNI material^27^ is also available with the proposed strategy under an extreme spinning velocity. The dominant component of the programmable manipulations presented in schemes 1–4 is the heat flux deflection at the fluid-bilayer interface under certain rotation rates. Such heat flux deflection can be also theoretically predicted based on Eqs. (1) and (2). It is noted that the effectively directional components in the Cartesian system are anisotropic due to the spinning effects.

**Fig. 2:**
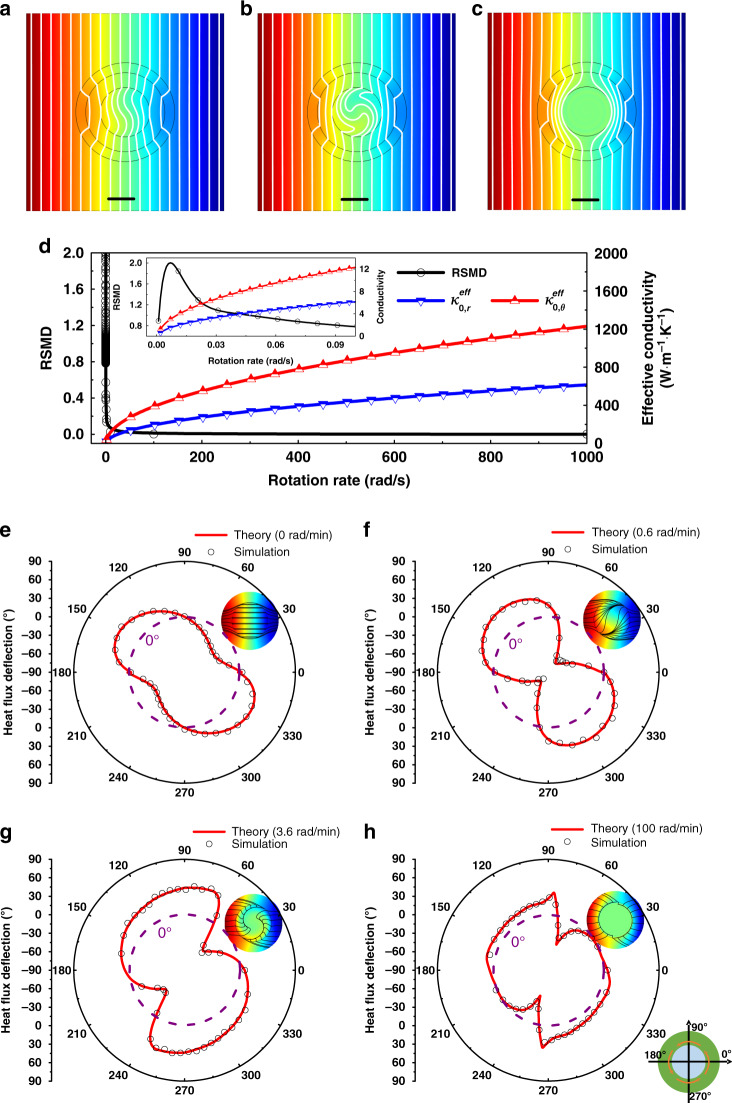
Temperature fields of the schemes with effective static solid centers and heat flux deflection at the spinning fluid interface along the structural azimuths. **a**–**c** respectively present the temperature fields with the effective static solid centers of actual fluid schemes 2–4 (Fig. 1d–f), whose conductive components follow the Supplementary Equation 21. The scale bar is 30 mm (black lines); **d** illustrates the effectively directional conductivities and RMSD between the actual and effective schemes. The effectively conductive components are enhanced by the increasing spinning velocities, thus contributing to the switchable field manipulations with tunable finite conductivities. Besides, effectively infinite conductivities can be also anticipated once the spinning velocity approaches infinity. Source data are provided as a Source Data file. **e**–**h** respectively denote the heat flux deflection of each scheme. The continuous lines are the solutions of Eq. (3), while the scatters denote direct simulated results. The dotted lines show the locations of 0°. The right upper insets show the heat flux distribution at the interface, and the right bottom inset illustrates the azimuths of physical structures.

To further validate the tunable analogs, additional cases of schemes 2–4 are implemented using an effective static solid center, where the spinning fluid domain is replaced by an anisotropic static solid domain with certain axis rotations to the principle system (see Supplementary Note 5). The temperature distributions of these cases with effective static solid centers are shown in Fig. 2a–c. Compared with the findings shown in Fig. 1c–f, the effective field distributions are quite approximate to the actual fluid domains, which indicates the accuracy of the effectively directional conductivities. Note that, the realization of such complicated conductive components of the contrasts with effective static solid centers reveals the large amounts of fragmented conductive elements^26^ in designing the practical devices. Besides, the structural changes are also inescapable in adjusting the locally conductive configurations for achieving the satisfied tunability. Compared with the above inconveniences of static solid schemes, the employment of fluid provides the elegant solution of realizing the expected tunability. Owing to the fluid continuity and mobility, the inhomogeneous solid functional region could be significantly simplified by a rotating fluidic domain, and the modulated velocities bring great conveniences in operations without any structural changes.

The values of Eqs. (1) and (2) at 45° are selected at different rotation rates and illustrated in Fig. 2d. Both the directional values increased with increasing rotation rates. Furthermore, arbitrary conductive components can be observed with specific rotation rates, while the effectively infinite conductivity can be also anticipated once the angular velocity approaches infinity. Equations (1) and (2) suggest that the directional conductivities can be expected to exceed the one of graphene (5300 W m^−1^ K^−1^), when the theoretical rotation rates are respectively larger than 18578 rad/min and 74314 rad/min. These high rotation rates can be anticipated by using an appropriate actuating motor with a high motor revolution (>100,000 rpm) and a related motion controller. If the enclosed region is fully filled with the target fluid^27^, one could also expect few effects of turbulence dissipation on the temperature profiles, since the thermal field would be rapidly homogenized by the extreme convections. In general, the tunability of conductive components ranges from the initial conductivity *κ*_0_ (*ω* = 0) to near infinity at the extreme spinning rate (*ω* → ∞). The root mean square derivations (RMSD) between the actual and effective schemes at varied rotation rates are calculated and illustrated in Fig. 2d. It indicates that almost zero temperature fluctuations can be observed in a broad range of rotation rates, which further validate the accuracy. Though some peaks and narrow fluctuant range are observed when the rotation rates are lower than 0.04 rad/min, such fluctuations are small enough. Hence, the spinning fluid can be used as a tunable analog thermal material under varied rotation rates.

Considering the field distributions and effectively local conductivities, the theoretical heat flux deflections at the spinning interface can be also obtained as shown in Fig. 2e–h (the red lines). Besides, simulated values are also illustrated as circular scatters to make a fair contrast. The simulated deflections overlap well with the solutions of Eq. (3). When the fluid is at rest, some initial heat flux deflections contributed by the regional conductive differences are observed at the interface as shown in Fig. 2e. As illustrated in Fig. 2f, g, the deflections of heat flux are dragged by the spinning fluid at low rotation rates, and the deformations at each azimuth become more severe with the increasing angular velocity. Further accelerating the fluid rotation, the deflections would gradually decrease and approach 0. That is, the respective deflections of the fluid domain and the internal layer are approximate, which can be canceled with each other. The maximum deflection would approach 45° at a large angular velocity^27^ as shown in Fig. 2h.

### Experimental demonstration of tunable analog thermal material

**Fig. 3:**
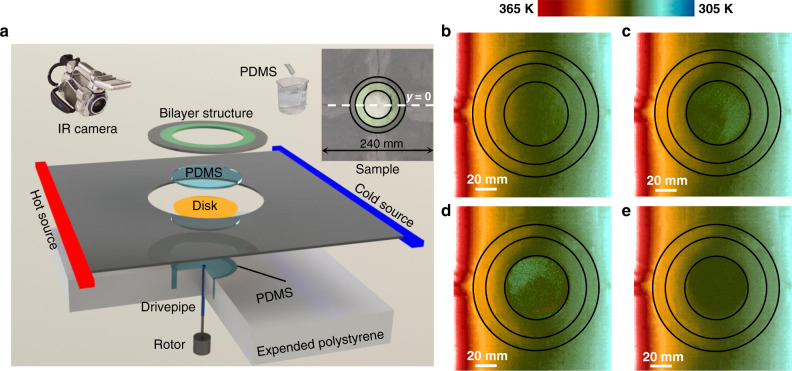
Experimental setups of the proposed scheme and measured temperature fields. **a** presents the general experimental setups and the fabricated sample. The white dashed line of *y* = 0 in the inset is the measured line for the thermal profiles in Fig. 4. The internal medium of the bilayer structure is a silicone pad annulus (*κ*_1_ = 1 W m^−1^ K^−1^), while the external layer is made of AISI 1010 steel annulus (*κ*_2_ = 51.9 W m^−1^ K^−1^). **b**–**e** denote the temperature distributions of the proposed schemes 1–4 at the rotation rates of *ω* = 0 rad min^−1^, *ω* = 0.6 rad min^−1^, *ω* = 3.6 rad min^−1^, and *ω* = 100 rad min^−1^.

To further demonstrate the proposed tunable analog thermal material via rotational excitation, we implement schematically experiments with the setups illustrated in Fig. 3a. The temperature distributions shown in Fig. 3b–e are measured by an infrared camera at the upper side of the system. The experimental field distributions are approximate to the simulated ones shown in Fig. 1c–f, which validate the significances of tunable analog thermal materials via the spinning system. The measured line of *y* = 0 mm is selected to quantitatively describe the modulated thermal manipulations.

**Fig. 4:**
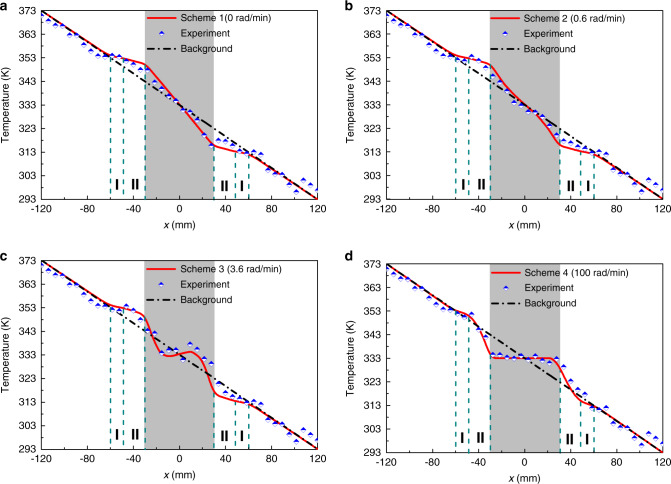
Experimental temperatures along the measured line of *y* = 0 mm (marked in the right upper inset of Fig. 3a. I and II respectively denote AISI 1010 steel annulus and silicone pad annulus, while the gray region is uncured PDMS. **a**–**d** illustrate the measured temperatures of schemes 1–4 under the rotation rates of *ω* = 0 rad min^−1^, *ω* = 0.6 rad min^−1^, *ω* = 3.6 rad min^−1^, and *ω* = 100 rad min^−1^.

The simulated and experimental temperature distributions at varied angular velocities along the measured line are illustrated in Fig. 4a–d. It indicates that both the simulated and experimental results overlap well, while significantly manipulative behaviors and non-distortion are respectively observed in the center and background. The temperatures in the backgrounds of each scheme approach to the distributions of a pure background, thus leading to the uniform temperature gradients without distortions. Such non-distortion fields in the background are contributed by the tailored bilayer structure, which is available to reshape the external field distributions under various velocities (see Supplementary Notes 1 and 5). Hence, the proposed method of a spinning system is available to create smooth fields at varied rotation rates, while a significant conductive tunability is simultaneously observed in the spinning center. When the fluid is at rest (Fig. 4a) or subject to high rotation rates (Fig. 4d), the temperature fields of enhanced transparency and sensitive cloaking can be achieved as in previous works. Also, the heat flux deflection can be flexibly manipulated with varied angular velocities such as the example of field contortion shown in Fig. 4b. Furthermore, once the angular velocity of *ω* = 3.6 rad·min^−1^ is excited as illustrated in Fig. 4c, the behavior of inverse field distribution can be realized without additional sources, where the trend of central temperature distribution is completely inversed to one in the background outside the bilayer structure. Such a system is able to achieve an inverted thermal propagation in the shaded region.

All the above behaviors showcase great potentials on active thermal manipulations. The proposed strategy of tunable analog thermal materials introduces the concept of dynamic thermal system into the wide range of thermal manipulations and related metamaterials. Compared with the conventional static thermal metamaterials, the long-existing challenges of complex medium configurations and structural switching can be significantly and elegantly solved by simply using a rotating fluid region with modulated velocities. Moreover, the wide range of modulating velocity further reveals an extensively and continuously conductive tunability and multifarious manipulations of heat flow control only in one single system. It is believed that such tunable analog thermal materials would further innovate the conventionally static thermal metamaterials, and provide the simpler and more flexible method of tunable field manipulations.

## Conclusion

We have proposed a new mechanism of tunable analog thermal material induced by a spinning component. Under the spinning effects of uncured PDMS, the continuously tunable conductivities/anisotropies ranging from inherent one to near-infinity can be easily achieved through coupling corresponding spinning rates without additional sources or structural adjustments. Meanwhile, some representative behaviors, including enhanced transparency, rotation, inverse field distribution, and sensitive cloaking, further reveal its significance. None of these features can be obtained in conventional thermal materials. The theoretical solution of effective conductivity and field deflection under general angular velocity may become the bond of stringing non-adjustable conductivities in a single material. Besides, the findings may also open an avenue for active and flexible thermal manipulation with the dynamic spinning system. It is believed that this would occupy an important role in motivating the current research on investigating and predicting heat transfer in moving matters.

## Methods

### Simulation of the schemes induced by spinning fluid

The entire bilayer structure induced by spinning fluid is set at the center of a 240 mm × 240 mm square background, which is made of a heat dissipation silicone pad with a thermal conductivity of *κ*_b_ = 12 W m^−1^ K^−1^. The external and internal radii of the bilayer structure are respectively set as 60 mm and 30 mm. Considering the matching relations shown in Supplementary Equation 10, a 12 mm width AISI 1010 steel annulus with a conductivity of *κ*_2_ = 51.9 W m^−1^ K^−1^ is selected as the external layer, and an 18 mm width silicone pad annulus with a conductivity of *κ*_1_ = 1 W m^−1^ K^−1^ is employed as the internal layer. For the center, uncured PDMS with an inherent conductivity of 0.15 W m^−1^ K^−1^ is filled to act as the spinning fluid. The reason for using uncured PDMS is its low enough conductivity and large enough viscosity. The low-conductivity contributes to the wide range of tunability (from near-zero to near-infinity), while the large enough viscosity makes it available to hold all fluid within as the convective role, and to significantly reduce the surface pressure and avoid the interrupted connections at arbitrary velocities. The simulation combined with heat conduction and convection is performed through Comsol Multiphysics 5.3a for the proposed schemes 1–4. During the simulated processes, the angular velocity fields with anticlockwise spinning rates of *ω* = 0 rad min^−1^, 0.6 rad min^−1^, 3.6 rad·min^−1^, and 100 rad min^−1^ are respectively inserted in the central domain of uncured PDMS. The left and right boundaries are respectively set as high-temperature wall (373 K) and low-temperature wall (293 K). The top and bottom boundaries are thermal insulations. The temperature configurations are sustained during the entire calculating processes.

### Simulation of the contrast schemes with effectively static solid centers

The temperature distributions shown in Fig. 2a–c are the contrasts of schemes 2~4 with the effectively static solid centers. The actual spinning fluid center used in the original schemes is replaced by effectively static solid objective, while the other parts keep unchanged. The conductive components in the effectively static solid centers of these contrasts strictly follow the findings of Supplementary Equation 21, and the related conductive components are directly inserted into the static solid center. The same boundary conditions are also employed in the contrasts. For the root mean square derivations (RMSD) between the actual fluid and effectively static solid central domains, the values can be calculated by the expression of ${\mathrm{RMSD}} = \frac{{{\sum} {\left( {T_{fluid} - T_{solid}} \right)^2} }}{n}$. Here, *T*_fluid_ and *T*_solid_ denote the temperatures of the calculated points respectively in the centers of spinning fluid schemes and effectively static solid cases, and *n* is the quantity of these calculated points. A series of simulations of the spinning fluid schemes at varied rotation rates and related contrast static solid cases are implemented accordingly.

### Experimental setups and demonstration

The entire system and fabricated sample are respectively illustrated in Fig. 3a and its upper inset. The background and bilayer structures are made of the abovementioned media and combined with each other. Due to the dimension limits of the actual heat dissipation silicone pad (*κ*_b_ = 12 W m^−1^ K^−1^), we spliced four independent heat dissipation silicone pads (120 mm × 120 mm) together into the background. To decrease the effects of thermal resistance, we filled thermally conductive silicone grease (12 W m^−1^ K^−1^) at the seams. The entire system is placed on an expanded polystyrene (EPS) foam. The core is the central region for inserting spinning fluid. The 30 mm thickness expanded polystyrene foam with a 3 mm depth circle hole (radial is 25 mm) in the center is employed to support the entire sample. A reserved channel with a depth of 25 mm and 2.5 mm thickness is drilled around the circle hole to gather extra fluid and prevent outflow. A disk (1 mm thickness) is set at the bottom of the hole for spinning the inserted fluid. The disk is connected to a rotor through the blue drive-pipe, and the drive-pipe is 1 mm higher than the bottom of the central hole. Under these operations, the rotational stability can be ensured and few outflows were observed during the entire experiment.

The high temperature of 373 K is imposed on the left side of the fabricated sample shown in Fig. 3 through a temperature-control heating strip (fixed at 373 K), while the right side is directly immersed into the room-temperature water (293 K). These temperature configurations are reserved and keep unchanged during the entire experiments and measurements, thus contributing to the constant thermal profiles in the background domain, and no additional structure or medium changes during the experiments. Since the emissivity of silicone pads approach 0.97, insulating tapes with an emissivity of 0.97 were employed to cover the upper surface of the center and the external metal layer to keep the similar emissivity of the entire system for IR imaging. The temperature distributions of the proposed schemes are captured by an IR camera (emissivity was set as 0.97), and the temperatures along the line of *y* = 0 mm were measured by thermocouples. The measurement is started with the first scheme of enhanced thermal transparency (*ω* = 0 rad min^−1^). The temperature field is captured after heating up 7200 s for achieving the steady-state thermal profiles. Then, the rotor is started and maintained at 0.6 rad min^−1^ for measuring the second scheme based on the field distribution of the first scheme. By that analogy, the third (*ω* = 3.6 rad min^−1^) and fourth (*ω* = 100 rad min^−1^) schemes are also implemented in turn.

It is worthy to note that the vertical distribution of uncured PDMS and viscous heating at arbitrary angular velocities have few effects on realizing the tunable conductivity. For the vertical distribution of uncured PDMS, both the reserved channel on the EPS foam and cover of insulated tape on the top of the 2-mm fluid layer would prevent the outflows under arbitrary velocities. Thus, the central domain can be regarded as an enclosed region always fulfilling with rotating fluids at arbitrary velocities, and the approximately flat fluid surface can be maintained without significantly vertical profile changes. For the aspect of viscous heating, its effect is not significant when the imposed velocity is small. Besides, the dissipation caused by the viscidity at large/extreme is far smaller than the components of inherent thermal conduction and convection inside the fluid domain^27^, thus contributing to the homogeneous thermal profile without temperature gradients under effectively large conductivity. Hence, viscous heating also has few effects on realizing tunable conductivity with rotating fluid, which can be neglected both at low and high velocities.

## Supplementary information

Supplementary Information

## Data Availability

The data that support the findings of this study are available from the corresponding authors on reasonable request. Source data are provided with this paper.

## Source data

Source Data
