# Supplementary material for: Tunable analog thermal material
Source: Nat Commun. 2020 Nov 27;11:6028. doi: 10.1038/s41467-020-19909-0 (PMC7699644; doi:10.1038/s41467-020-19909-0)
Supplement: Supplementary file 1 — Supplementary Information [file 41467_2020_19909_MOESM1_ESM.pdf]

Supplementary Information

Tunable analog thermal material

Xu et al.

Supplementary Note 1: Derivations of effective solid configuration in a bilayer structure

Considering the rotations of the actual fluid domain under finite angular velocity, the field deviation in the azimuthal direction should be considered at the solid-fluid interface. Hence, the entire system can be regarded as a solid bilayer system with an anisotropic center. To analyze the system, the rotated fluid center is considered as an effective solid plate with anisotropic conductivities of $\kappa_{0,r}^{eff}(\theta)$ and $\kappa_{0,\theta}^{eff}(\theta)$ along the principle axes (r and θ). To create expected behaviors in a solid system without external field distortion, a rigorous analysis based on steady-state heat conduction equation ($\nabla \cdot (-\kappa \cdot \nabla T) = 0$) is implemented in cylindrical coordinates for matching adjacent fields. The schematic of the bilayer system is shown in Supplementary Figure 1.

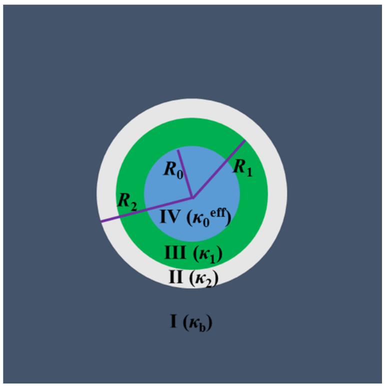

Supplementary Figure 1. Functional regions of a bilayer structure

Here, the following basic assumptions should be obeyed. For achieving a non-distortion temperature field in the external background ($r > R_2$), the temperature at the interface of background (I) and external layer (II) should be $-r\cos\theta \cdot \nabla T_I$ when r approaches infinity. Besides, the temperature in the tailored region is finite when r approaches zero. Under the above preconditions, the temperature and heat flux distributions at the interfaces of adjacent regions can be obtained. The general solutions of temperature distributions for each region shown in Supplementary Figure 1 can be expressed as follows:

$$T_{\mathrm{I}} = \left(Ar + Br^{-1}\right)\cos\theta. \quad \left(r > R_2\right). \tag{1}$$

$$T_{\mathrm{II}} = \left(Cr + Dr^{-1}\right)\cos\theta \quad \left(R_1 < r \le R_2\right). \tag{2}$$

$$T_{\mathrm{III}} = \left(Er + Fr^{-1}\right)\cos\theta \quad \left(R_0 < r \le R_1\right). \tag{3}$$

$$T_{\mathrm{IV}} = G_1\left(r\right)\cdot G_2\left(\theta\right) \qquad \left(r \le R_0\right). \tag{4}$$

In equations (1) ~ (4), $A \sim F$ are scaled components of the solutions, which can be derived from the boundary conditions. Due to the anisotropic and nonhomogeneous conductivities of the center, the temperature of region IV cannot be directly presented with the standard form of Laplace equation. Hence, the components of implicit

functions $G_1(r)$ and $G_2(\theta)$ are employed to describe the temperature distribution in the center. It's noted that B should be 0 under the above assumption. r denotes the specific locations. Under certain temperature gradient ∇T_I along radial direction generated at the system boundary, the field distribution in each region can be obtained with the temperature and flux consistencies at adjacent boundaries. Here, the boundary conditions at the regional interfaces can be written as:

At the interface of $r = R_2$:

$$\begin{cases} A \cdot R_2^2 + B - C \cdot R_2^2 - D = 0, \\ \kappa_b A \cdot R_2^2 - \kappa_b B - \kappa_2 C \cdot R_2^2 + \kappa_2 D = 0. \end{cases} \tag{5}$$

At the interface of $r = R_1$:

$$\begin{cases} C \cdot R_1^2 + D - E \cdot R_1^2 - F = 0, \\ \kappa_2 C \cdot R_1^2 - \kappa_2 D - \kappa_1 E \cdot R_1^2 + \kappa_1 F = 0. \end{cases} \tag{6}$$

Due to the assumption of an effective solid domain of center under the finite angular velocity with rotated fluid, the heat conduction inside region III follows the steady-state heat conduction equation ($\nabla \cdot (-\kappa_1 \cdot \nabla T_1) = 0$) for isotropic medium, while the effective heat transfer inside region IV should obey the governing function of $\frac{\partial}{r\,\partial r} \cdot \left(r\kappa_{0,r}^{eff}(\theta) \cdot \frac{\partial T_0}{\partial r} \right) + \frac{\partial}{r\,\partial \theta} \cdot \left(\kappa_{0,\theta}^{eff}(\theta) \cdot \frac{\partial T_0}{r\,\partial \theta} \right) = 0$ for anisotropic medium[1]. The general solution of the central temperature can be obtained with $G_1(r)$ and $G_2(\theta)$, which can be expressed as follows:

$$\frac{\partial^2 \left(G_1(r) \right)}{\partial r^2} = \frac{G_1(r)}{r^2} - \frac{\partial \left(G_1(r) \right)}{r \cdot \partial r} \qquad \left(r \le R_0 \right). \tag{7}$$

$$\frac{\partial^2 \left(G_2(\theta) \right)}{\partial \theta^2} = -G_2(\theta) \cdot \frac{\kappa_{0,r}^{eff}(\theta)}{\kappa_{0,\theta}^{eff}(\theta)} - \frac{\partial \left(\kappa_{0,\theta}^{eff}(\theta) \right)}{\kappa_{0,\theta}^{eff}(\theta) \partial \theta} \cdot \frac{\partial \left(G_2(\theta) \right)}{\partial \theta} \qquad \left(r \le R_0 \right) \tag{8}$$

Considering the assumption of finite temperature of the center, the general solution of equation (7) can be expressed as: $G_1(r) = G \cdot r$. Hence, the thermal field at the interface of $r = R_0$ can be expressed as:

$$\begin{cases} \left(E \cdot R_0^2 + F \right) \cos(\theta) - R_0 \cdot G_1\left(R_0 \right) \cdot G_2(\theta) = 0, \\ \left(\kappa_1 E \cdot R_0^2 - \kappa_1 F \right) \cos(\theta) - \kappa_{0,r}^{eff}(\theta) \cdot R_0^2 \cdot G_1'\left(R_0 \right) \cdot G_2(\theta) = 0. \end{cases} \tag{9}$$

Solving equation (9), the relations among the constants of E, F, and G can be achieved. Considering the temperature gradient ∇T_I along radial direction and conductivities of the system under the assumption of $B = 0$, the constants $A \sim F$ and the component functions $G_1(r)$ and $G_2(\theta)$ can be achieved. Furthermore, the following principle of matching adjacent field and avoiding external distortions can be derived by combining the scaled components.

$$
\begin{aligned}
&\left(\left(\kappa_1+\kappa_{0,r}^{eff}(\theta)\right)\cdot\left(\kappa_2-\kappa_1\right)\cdot R_1^2+\left(\kappa_1-\kappa_{0,r}^{eff}(\theta)\right)\cdot\left(\kappa_1+\kappa_2\right)\cdot R_0^2\right)\cdot\left(\kappa_2+\kappa_b\right)\\
&=\left(\left(\kappa_1+\kappa_{0,r}^{eff}(\theta)\right)\cdot\left(\kappa_1+\kappa_2\right)\cdot R_2^2+\left(\kappa_1-\kappa_{0,r}^{eff}(\theta)\right)\cdot\left(\kappa_2-\kappa_1\right)\cdot\frac{R_0^2R_2^2}{R_1^2}\right)\cdot\left(\kappa_2-\kappa_b\right)
\end{aligned}
\tag{10}
$$

Supplementary Note 2: Effective conductivity at the solid-fluid interface

Boundary conditions of the effective solid bilayer structure

First of all, we derivate effective values at the interface between regions III and IV. The boundary conditions of effective temperature and heat flux at the solid-fluid interface ($r = R_0$) can be written with constant G:

$$
\begin{cases}
T_1^{eff}\big|_{r=R_0}=T_0^{eff}\big|_{r=R_0}=\left(E\cdot R_0+F\cdot R_0^{-1}\right)\cos(\theta)=GR_0\cdot G_2(\theta),\\
\kappa_1\dfrac{\partial T_1^{eff}}{\partial r}\bigg|_{r=R_0}=\kappa_{0,r}^{eff}\dfrac{\partial T_0^{eff}}{\partial r}\bigg|_{r=R_0}=\kappa_{0,r}^{eff}\cdot G\cdot G_2(\theta).
\end{cases}
\tag{11}
$$

In equation (11), T_1^{eff} and T_0^{eff} denote the effective temperatures at the interface between regions III and IV.

Boundary conditions of the actual fluid center

For matching the effective solid domain and rotated fluid, the same principle system is considered here. Then, the actual temperature distributions of the fluid domain ($r < R_0$) at steady state can be also achieved with the following convective equation:

$$
\frac{\kappa_0}{\rho_0 c_0}\left(\frac{\partial^2 T}{\partial r^2}+\frac{1}{r}\frac{\partial T}{\partial r}+\frac{1}{r^2}\frac{\partial^2 T}{\partial\theta^2}\right)=\omega\frac{\partial T}{\partial\theta}\quad\left(r\le R_0\right).
\tag{12}
$$

In equation (12), κ_0 is the initial conductivity of the employed fluid. As [2] indicated, the general solution of the above equation can be obtained with the first order Kelvin's function. Hence, the actual temperature distribution inside the fluid domain can be expressed as:

$$
T_0(r,\theta)=M(r)\cdot\cos\left(\theta-\phi(\omega,r)\right)\qquad\left(r\le R_0\right).
\tag{13}
$$

In equation (13), $\phi(\omega, r)$ is an argument in the solution to illustrate the rotational effect, and it is a continuous real function of ω and r, which can be defined in the general solution and calculated in the convective process. According to the properties of Bessel function, the general temperature gradients at radial directions can be written as follows:

$$
\frac{\partial T_0}{\partial r}=\sqrt{\frac{\omega\rho c}{\kappa}}\cdot M(r)\cdot\cos\left(\theta-\phi(\omega,r)+\frac{\pi}{4}\right).
\tag{14}
$$

As indicated in [2], θ-$\phi(\omega)$+$\pi/4$ should approach θ based on minimum entropy production, when ω is large enough. In this paper, the general case with modulated angular velocity is considered. Hence, the specific condition used at high speed should be motivated to a general one. It indicates that a larger temperature gradient

can be obtained at a low angular velocity of fluid flow. That is, a finite thermal field deflection under the prerequisite of minimum entropy production is observed. Hence, the initial relation of $\phi(\omega, r) = \phi\left(\sqrt{\frac{\omega\rho c}{\kappa}} r\right) = \arctan\left(bei_1\left(\sqrt{\frac{\omega\rho c}{\kappa}} r\right)/ber_1\left(\sqrt{\frac{\omega\rho c}{\kappa}} r\right)\right)$ should be employed here[3]. According to the asymptotic expansions under the condition of $\sqrt{\frac{\omega\rho c}{\kappa}} r \to \infty$, the value of $\phi\left(\sqrt{\frac{\omega\rho c}{\kappa}} r\right)$ approaches π/4.

Boundary coupling of the effective solid domain and actual fluid system

The next step is to couple the effective and actual values at the interface (same location). Since the effectively anisotropic solid domain is employed to make an equivalence of the actual fluid, its locally effective values at the boundary $r = R_0$, including the temperature and radial heat flux, should be in accordance with the actual fluid on each point. Hence, the following relations at the boundary ($r = R_0$) should be obeyed for a general case:

$$\begin{cases} T_0^{eff}\Big|_{r=R_0} = T_0\Big|_{r=R_0}, \\ \kappa_{0,r}^{eff}(\theta)\dfrac{\partial T_0^{eff}}{\partial r}\Big|_{r=R_0} = \kappa_0 \dfrac{\partial T_0}{\partial r}\Big|_{r=R_0} \end{cases}. \tag{15}$$

Whether the actual fluid domain or the effective solid plate, the same interface of $r = R_0$ is shared. Hence, both of the actual and effective temperatures on each point along the interface should be same. However, this condition cannot be achieved under a spinning system, due to the azimuthal field deformation. Hence, the differences between the actual and effective values on each point should be minimum.

$$\begin{aligned} \int_0^{2\pi}\left(T_0\Big|_{r=R_0} - T_0^{eff}\Big|_{r=R_0}\right)^2 d\theta &= \int_0^{2\pi}\left(M(r)\cdot\cos\left(\theta-\phi(\omega,r)\right) - GR_0\cdot G_2(\theta)\right)^2 d\theta \\ &= \pi\left(M(R_0)\right)^2 + GR_0\int_0^{2\pi} G_2(\theta)\left(GR_0 G_2(\theta) - 2M(R_0)\cdot\cos\left(\theta-\phi(\omega,r)\right)\right)d\theta \end{aligned} \tag{16}$$

Considering the diffusive system, the first order of the solution $G_2(\theta)$ can be employed here to simplify equation (16), thus approximately leading to the following relation:

$$\int_0^{2\pi}\left(T_0\Big|_{r=R_0} - T_0^{eff}\Big|_{r=R_0}\right)^2 d\theta = \pi\left(M(R_0)\cdot\cos\left(\phi(\omega,r)\right) - GR_0\right)^2 + \pi\left(\left(M(R_0)\cdot\sin\left(\phi(\omega,r)\right)\right)^2\right) \tag{17}$$

The minimized temperature deviation can be achieved under specific angular velocity, once the following relation is matched.

$$M(R_0)\cdot\cos\left(\phi(\omega,r)\right) = GR_0 \tag{18}$$

Taking equation (18) into the boundary conduction of equation (15), the locally effective radial conductivity at the solid-fluid interface can be obtained.

$$\kappa_{0,r}^{eff} = R_0\sqrt{\omega\rho c\kappa_0}\cdot\frac{\cos\left(\theta-\phi(\omega,r)+\frac{\pi}{4}\right)}{\cos(\phi(\omega,r))\cos(\theta)}. \tag{19}$$

To keep the equivalence of effective solid and actual fluid systems, the component $\kappa_{0,\theta}^{eff}(\theta)$ is employed to describe the field deflections at azimuthal directions in an effective solid system, which are caused by the convection of the actual fluid. Taking equation (19) into the governing function $\frac{\partial}{r\,\partial r}\cdot\left(r\kappa_{0,r}^{eff}(\theta)\cdot\frac{\partial T_0}{\partial r}\right)+\frac{\partial}{r\,\partial\theta}\cdot\left(\kappa_{0,\theta}^{eff}(\theta)\cdot\frac{\partial T_0}{r\,\partial\theta}\right)=0$ for the effective central region, the locally effective azimuthal conductivity can be expressed as follow.

$$\kappa_{0,\theta}^{eff} = 2R_0\sqrt{\omega\rho c\kappa_0}\cdot\frac{\sin\left(\theta-\phi(\omega,r)+\frac{\pi}{4}\right)}{\cos(\phi(\omega,r))\sin(\theta)}. \tag{20}$$

Different from the previous work on KNI medium[2] at extremely large velocity, the azimuthal component of effective conductivity (equation (20)) is existed here for matching the azimuthal field deflection at a general angular velocity. Such component is based on the derivations of the azimuthal temperature gradients between the effective domain and actual fluid at a general velocity, i.e., $\theta-\phi(\omega, r)+\pi/4\neq\theta$. It would also approach infinity once the rotational excitation is large enough. Thus, the temperature inside the fluid domain would be rapidly homogenized by the convective effects with a high local heat transfer coefficient, which leads to the temperature response verse time of the KNI medium. For the case at a general velocity, the azimuthal component is finite and higher than the radial one. Such conductive components contribute to the field deflections of the fluid domain. In addition, the deflection would be enhanced with increasing angular velocity, which leads to the torsional fields shown in Figs. 1 and 3.

In general, equations (19) and (20) provide the conductive distributions at the solid-fluid interface. The directional conductivities at the boundary ($r = R_0$) of the central domain are presented in Supplementary Figure 2. The extreme values of radial conductivities $\kappa_{0,r}^{eff}$ can be obtained at the azimuths of 90° and 270°, while the extreme $\kappa_{0,\theta}^{eff}$ would be achieved at the locations of 0° and 180°. The reason for the extreme values is the parallel directions to the principle axes (r and θ) of local heat transfer processes at these locations. Owing to the axis rotation to the principle system in the schemes of the effective static solid center, such extreme values are suppressed. Moreover, the values of each directional conductivity increase with the increasing rotation rates, and the azimuthal conductivity is higher than the radial one. Due to the inhomogeneous directional conductivities, the

thermal fields could exhibit the rotational temperature distributions. Further increasing the rotation rates, such rotational distributions are enhanced with more homogenized temperatures, which would be beneficial to create the behavior of sensitive cloaking with few temperature gradients. Furthermore, the directional conductivities approach homogeneous in a wide range of azimuths, except the locations near the azimuths along the principle axes. Hence, the entire changing trends of $\kappa_{0,r}^{eff}$ and $\kappa_{0,\theta}^{eff}$ at varied rotation rates can be indicated by the homogeneous values. Thus, the values of $\kappa_{0,r}^{eff}$ and $\kappa_{0,\theta}^{eff}$ at 45∘ are selected at different rotation rates and illustrated in Fig. 2d of the manuscript. Both the values increased with the increasing rotation rates. Moreover, arbitrary conductive components can be observed with a specific rotation rate, while the effectively infinite conductivities can be also observed once the angular velocity reaches infinity.

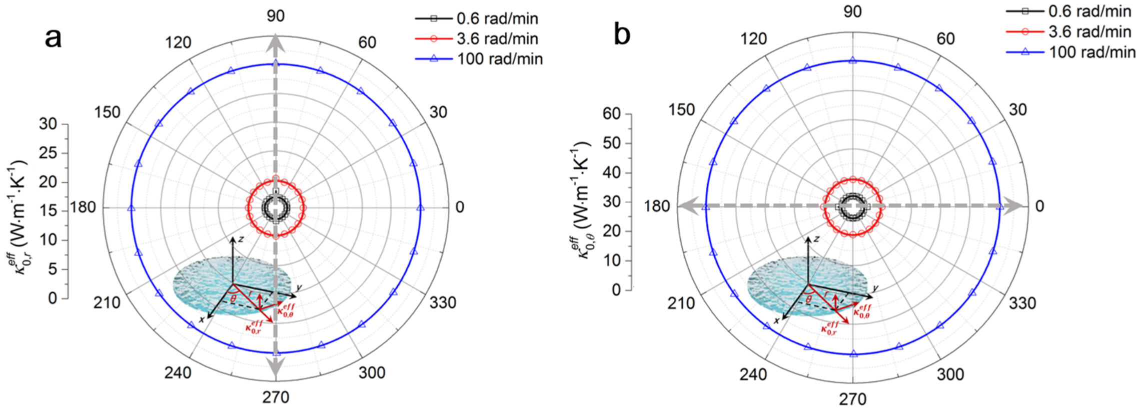

Supplementary Figure 2. Effectively directional conductivities of actual fluid schemes 2 ~ 4. **a** denotes the effectively radial components; **b** presents the effectively azimuthal conductivities.

Effect of inhomogeneous flow on thermal profiles

The rotating fluid actually leads to the inhomogeneous flow in the modulations. Such inhomogeneous flow further contributes to the deflected temperature fields under various velocities in the fluidic center as illustrated in Supplementary Figure 3. It is obvious that uniform isotherms and heat flux can be obtained without convection (Supplementary Figure 3a). When excited velocities are imposed, they become inhomogeneous and gradually exhibit rotating distributions with the increasing velocities (Supplementary Figures 3b ~ d). These demonstrations at modest velocities indicate that the temperature fields are dragged by the rotating flow, and the deflected intensity enhances with the increasing angular velocities. Besides, non-zero fluid temperature gradients are also observed at modest velocities, which further reveal the non-cloaking behaviors.

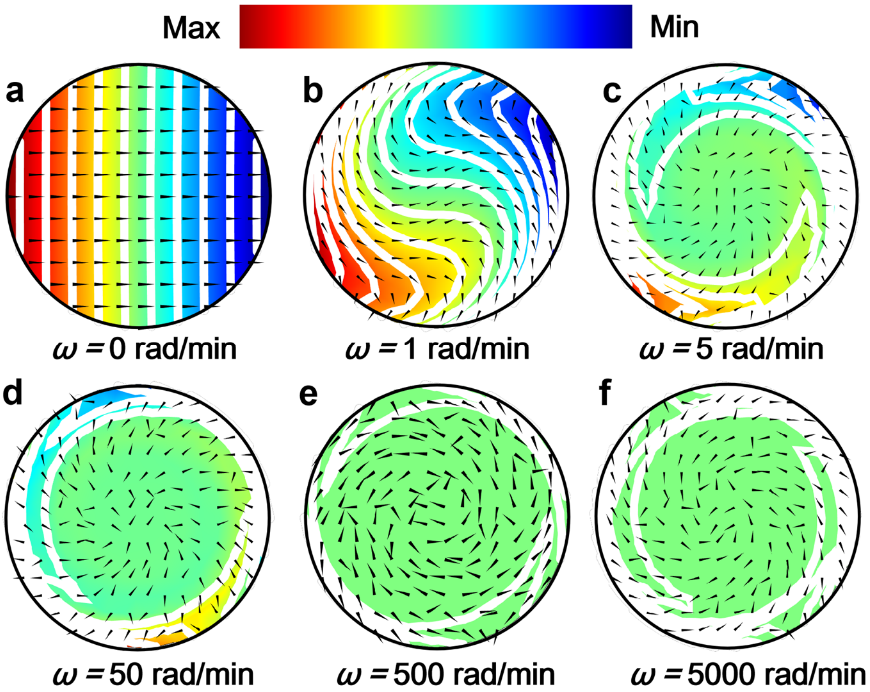

Supplementary Figure 3. Effects of the inhomogeneous flow on the distributions of thermal profiles and heat fluxes in the fluid center. The white lines and black arrows respectively denote isotherms and heat fluxes.

Though the temperature differences are quite small in Supplementary Figures 3e and f, the rotating isotherms and heat fluxes can be also observed at high/extreme velocities. In other words, the field distributions under extreme velocities actually also exhibit inhomogeneous forms, though quite small temperature gradients can be neglected. It is thus indicating that the homogeneous description of effective conductivity in [2] is an approximation under extreme convections without considering the effects of inhomogeneous flow on thermal profiles. When evaluating the thermal behaviors at moderate velocities, such effects of inhomogeneous flow should be involved to describe the deflected temperature fields and multifarious functions (not limited to cloaking).

Effect of fluid viscidity

The fluid viscidity would affect the practical operations of tunable analog thermal materials. Here, we respectively employ the uncured PDMS and water in the center to make a fair comparison of its effects at a moderate velocity. As shown in Supplementary Figure 4, the surface pressure of the uncured PDMS is much lower than that of water at the same moderate-velocity, which reveals fewer outflows along radial direction with large viscosity fluid. This could help uncured PDMS to hold the fluid firmly within when mechanical rotation is employed, and further maintain the uninterrupted connections between the boundaries of uncured PDMS and surrounding solids. Hence, more robust fluid rotations can be achieved at arbitrary velocities.

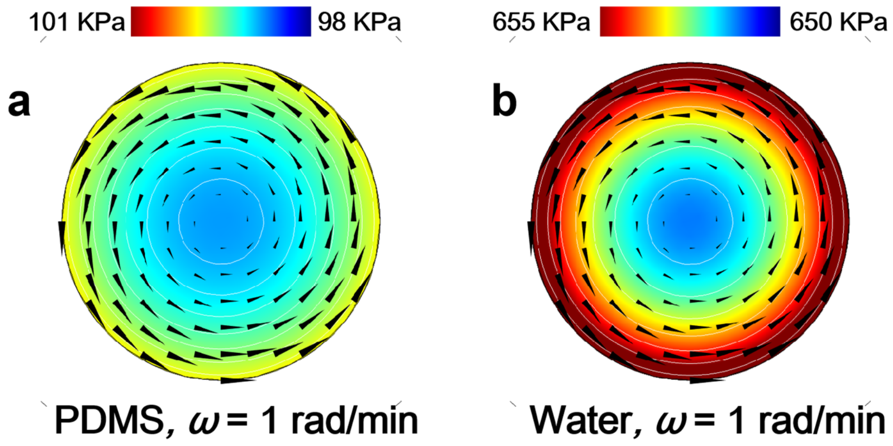

Supplementary Figure 4. Surface pressures of the fluid center respectively filled with uncured PDMS and water at the same moderate velocity. The black arrows denote the velocity distributions. The initial pressure of the fluid is 101 KPa.

Effective static solid center

Upon the effective conductivity along the internal boundary of the actual fluid domain, we indicate that the entire fluid center can be further considered as an anisotropic static solid domain with an axis rotation to the principle system (r, θ). Under the consideration of spinning effects of actual fluid, the system rotation to the principle system (r, θ) caused by varied rotation rates $\phi\left(\sqrt{\frac{\omega\rho c}{\kappa}}r\right)$ should be also involved in the effective solid center. As mentioned above, $\phi\left(\sqrt{\frac{\omega\rho c}{\kappa}}r\right)$ approaches $\pi/4$ once $\sqrt{\frac{\omega\rho c}{\kappa}}r \rightarrow \infty$, while it changes continuously with the increasing rotation rates. Besides, the temperature gradient would deflect round and round with the increasing rotation rates, and become stable once the rotation rate is large enough in the rotated fluid domain. During this process, the deflection $\phi\left(\sqrt{\frac{\omega\rho c}{\kappa}}r\right)$ would reach $\pi/4$ (final stable azimuth) several times with the increasing rotation rates. That is, the system rotation in the effective static solid system can be considered as the superposition time of reaching $\pi/4$ at varied rotation rates of the actual fluid domain. Hence, the effective static solid system (r', θ') can be expressed as[4]: $r' = r,\ \theta' = \theta + \frac{r}{R_0}\left(\frac{\pi}{4}\sqrt{\frac{\omega}{\omega_1}}\right)$. Here, ω_1 denote the rotation rate when the system rotation $\phi\left(\sqrt{\frac{\omega\rho c}{\kappa}}r\right)$ first reaches $\pi/4$. In the current system, the value of ω_1 is 0.003 rad/s.

Taking the effective static solid system (r', θ') into equations (19) and (20), the conductive components in the effective system, which is used to achieve field distributions of effective static solid center shown in Figs. 2a ~ c can be obtained.

$$\kappa'_{\text{solid},eff} = \begin{bmatrix} \kappa_{0,r}^{eff} & \frac{r}{R_0}\cdot\left(\frac{\pi}{4}\sqrt{\frac{\omega}{\omega_1}}\right)\cdot\kappa_{0,r}^{eff} \\ \frac{r}{R_0}\cdot\left(\frac{\pi}{4}\sqrt{\frac{\omega}{\omega_1}}\right)\cdot\kappa_{0,r}^{eff} & \left(\frac{r}{R_0}\cdot\left(\frac{\pi}{4}\sqrt{\frac{\omega}{\omega_1}}\right)\right)^2\cdot\kappa_{0,r}^{eff}+\kappa_{0,\theta}^{eff} \end{bmatrix}. \tag{21}$$

The temperature distributions of the effective schemes with effectively static solid centers shown in Figs. 2a ~ c are based on the above conductive components. To further verify the accuracy of the effectively directional conductivity and the effectively static solid center, the temperatures in the schemes with actual fluid and effectively static solid central domains are selected to make a fair comparison. Here, root mean square derivations (RMSD) between the actual fluid and effectively static solid central domains at varied rotation rates, i.e., $\mathrm{RMSD} = \sqrt{\frac{\Sigma(T_{fluid}-T_{solid})^2}{n}}$, are calculated to present the fluctuations. The results of RMSD are also illustrated in Fig. 2d of the manuscript. It indicates that almost zero temperature fluctuations can be observed in a broad range of rotation rates, which further validates the accuracy of the effectively directional conductivity and the effectively static solid center. Some peaks are observed when the rotation rates are lower than 0.04 rad/min, which are caused by the approximation of equation (17) and the dramatic changing $\phi\left(\sqrt{\frac{\omega\rho c}{\kappa}}r\right)$ at a low rotation rate. Fortunately, such fluctuations are small enough, as the largest RMSD is about 2. Furthermore, the range of fluctuations is also quite narrow. That is, the effectively directional conductivity can be employed to accurately describe the field distributions of an effectively static anisotropic domain. Hence, the spinning fluid can be used as the tunable analog thermal material under varied rotation rates.

Supplementary Note 3: Local heat flux deflection at the solid-fluid interface

Since the field deflection is the dominating component of manipulations, we further investigate the heat flux deflection at the interface. Taking the internal layer and center as an entire block, the effective conductivity can be observed based on effective medium theory in principle system[5]:

$$\begin{cases} \kappa_{\text{block},r}^{eff}(\theta,\omega) = \dfrac{f_1\kappa_{1,r}+f_0\kappa_{0,r}^{eff}}{f_0+f_1}, \\ \kappa_{\text{block},\theta}^{eff}(\theta,\omega) = \dfrac{\kappa_{1,\theta}\cdot\kappa_{0,\theta}^{eff}}{f_0\kappa_{1,\theta}+f_1\kappa_{0,\theta}^{eff}}. \end{cases} \tag{22}$$

Considering the heat transfer between inhomogeneous solid media[5], the heat flux deflection at the interface can be achieved with the heat flux components along x and y directions. The effective conductivity at the solid-fluid interface in Cartesian coordinate can be written as:

$$\begin{bmatrix} \kappa_{0,xx}^{eff}(\theta,\omega) & \kappa_{0,xy}^{eff}(\theta,\omega) \\ \kappa_{0,yx}^{eff}(\theta,\omega) & \kappa_{0,yy}^{eff}(\theta,\omega) \end{bmatrix} = \begin{bmatrix} \dfrac{\partial r}{\partial x} & \dfrac{\partial r}{\partial y} \\ \dfrac{\partial \theta}{\partial x} & \dfrac{\partial \theta}{\partial y} \end{bmatrix} \cdot \begin{bmatrix} r^2 \kappa_{\text{block},r}^{eff}(\theta,\omega) & 0 \\ 0 & r^2 \kappa_{\text{block},\theta}^{eff}(\theta,\omega) \end{bmatrix} \cdot \begin{bmatrix} \dfrac{\partial r}{\partial x} & \dfrac{\partial \theta}{\partial x} \\ \dfrac{\partial r}{\partial y} & \dfrac{\partial \theta}{\partial y} \end{bmatrix}. \tag{23}$$

Considering the constant temperature gradient along x direction in this work, the heat flux components can be expressed as:

$$\begin{cases} q_x = -\kappa_{0,xx}^{eff}(\theta,\omega) \cdot \dfrac{\partial T}{\partial x}, \\ q_y = -\kappa_{0,yx}^{eff}(\theta,\omega) \cdot \dfrac{\partial T}{\partial x}. \end{cases} \tag{24}$$

In equation (24), q_x and q_y are the components of heat flux along the temperature gradient. From the view of the local system along the temperature gradient, $\kappa_{0,xx}^{eff}$ and $\kappa_{0,yx}^{eff}$ can be also considered as the directionally conductive components in the local system, as only constant temperature gradient along x direction is excited in the entire system. Hence, the effective heat flux at an arbitrary azimuth of the interface in the local system can be written as[5]:

$$\begin{aligned} \begin{pmatrix} q_x^\theta \\ q_y^\theta \end{pmatrix} &= \begin{pmatrix} \cos\theta & -\sin\theta \\ \sin\theta & \cos\theta \end{pmatrix} \begin{pmatrix} \kappa_{0,xx}^{eff}(\theta,\omega) & 0 \\ 0 & \kappa_{0,yx}^{eff}(\theta,\omega) \end{pmatrix} \begin{pmatrix} \cos\theta & \sin\theta \\ -\sin\theta & \cos\theta \end{pmatrix} \begin{pmatrix} \dfrac{\partial T}{\partial x} \\ 0 \end{pmatrix} \\ &= \begin{pmatrix} \kappa_{0,xx}^{eff}(\theta,\omega)\cdot\cos^2\theta + \kappa_{0,yx}^{eff}(\theta,\omega)\cdot\sin^2\theta & \left(-\kappa_{0,xx}^{eff}\cdot(\theta,\omega) + \kappa_{0,yx}^{eff}\cdot(\theta,\omega)\right)\cos\theta\sin\theta \\ \left(-\kappa_{0,xx}^{eff}\cdot(\theta,\omega) + \kappa_{0,yx}^{eff}\cdot(\theta,\omega)\right)\cos\theta\sin\theta & \kappa_{0,xx}^{eff}(\theta,\omega)\cdot\sin^2\theta + \kappa_{0,yx}^{eff}(\theta,\omega)\cdot\cos^2\theta \end{pmatrix} \begin{pmatrix} \dfrac{\partial T}{\partial x} \\ 0 \end{pmatrix}. \end{aligned} \tag{25}$$

where, q_x^θ and q_y^θ are the heat flux components in the local coordinate system. θ is the azimuthal component on the line of $r = R_0$. The heat flux deflection at the interface can be achieved with the consideration of local azimuths:

$$\theta_{\text{bend}} = \arctan\left(\frac{q_y^\theta}{q_x^\theta}\right) = \arctan\left(\frac{\left(-\kappa_{0,xx}^{eff}(\theta,\omega) + \kappa_{0,yx}^{eff}(\theta,\omega)\right)\cos\theta\sin\theta}{\kappa_{0,xx}^{eff}(\theta,\omega)\cdot\cos^2\theta + \kappa_{0,yx}^{eff}(\theta,\omega)\cdot\sin^2\theta}\right). \tag{26}$$

Supplementary Note 4: Field distribution and heat flux deflections under large spinning velocity

To further validate the effective conductive components under high spinning velocity, an additional case with the spinning velocity of 1000 rad/min is proposed here. The temperature distribution and heat flux deflections at the regional boundary are shown in Supplementary Figure 5. The sensitive cloaking behaviors can be also observed at this stage owing to the effectively large conductivities. The heat flux deflections illustrated in Supplementary Figure 5b indicates that the maximum deflection would also approach 45°, while the values at most of the azimuths are near zero. These behaviors overlap well with the findings of the main contents, and the effectively infinite conductivities can be anticipated when the spinning velocity approaches infinity. It's

noteworthy that the behavior of sensitive cloaking can be observed in the cases with a wide range of spinning velocities, since the effectively large conductivity contributes to the rapid homogenization of thermal profiles with few temperature gradients.

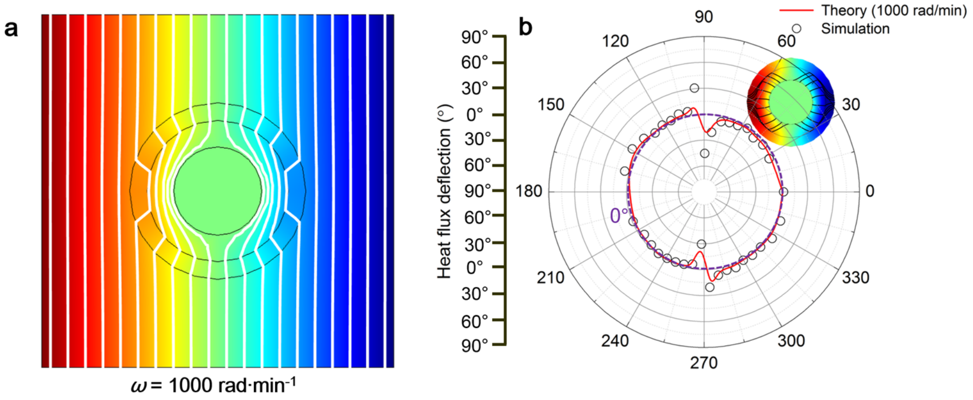

Supplementary Figure 5. Temperature distributions and heat flux deflections at the regional boundary of the case at 1000 rad/min. **a** illustrates the temperature distributions; **b** denotes its heat flux deflections.

Supplementary Note 5: Illustrations on the non-distortion field at varied angular velocities

The significant transparency with enhanced gradients shown in Figs. 1c and 3b are directly contributed by the matching relations shown in equation (10), as the near-zero conductivity κ_0 can be ignored. Hence, the matching condition degrades into the one independent of central conductivity:

$$\left(\left(\kappa_2-\kappa_1\right)\cdot R_1^2+\left(\kappa_1+\kappa_2\right)\cdot R_0^2\right)\cdot\left(\kappa_2+\kappa_b\right)=\left(\left(\kappa_1+\kappa_2\right)\cdot R_2^2+\left(\kappa_2-\kappa_1\right)\cdot\frac{R_0^2 R_2^2}{R_1^2}\right)\cdot\left(\kappa_2-\kappa_b\right). \quad (27)$$

The significances of active manipulations under low angular velocities without field distortion shown in Figs. 1d, 1e, 3c, and 3d are also contributed by equation (10). The matching condition at low angular velocity can be also approximate to the ones independent of the fluid, as low speed would provide a close value of $\kappa_{0,r}^{eff}$ to the constant κ_1. That is, the component including $-\kappa_{0,r}^{eff}$ approaches zero while the non-zero one with $+\kappa_{0,r}^{eff}$ can be eliminated in equation (10). Thus, the non-distortion field can be ensured at a low angular velocity, as the designed parameters follow the modified matching condition.

$$\left(\kappa_2-\kappa_1\right)\cdot R_1^2\cdot\left(\kappa_2+\kappa_b\right)=\left(\kappa_1+\kappa_2\right)\cdot R_2^2\cdot\left(\kappa_2-\kappa_b\right). \quad (28)$$

For the sensitive cloaking behavior at high angular velocity as shown in Figs. 1f and 3e, the component including effective $\kappa_{0,r}^{eff}$ can be eliminated in equation (10), due to the far larger non-zero values $\kappa_{0,r}^{eff}$ than the other regional conductivities. Thus, the matching condition at high angular velocity is in accordance with the

abovementioned one (equation (27)) without regional rotation of enhanced transparency case.

Supplementary Note 6: Differences between the tunable analog thermal material and thermal ground plane

We are aware that the technique of thermal ground plane (TGP) can be also used to achieve the effectively extreme-large conductivity and rapidly passive heat spreading. Though there exists a similarity of the effectively extreme-large conductivity, the TGP and tunable analog thermal materials can be regarded as two independent techniques respectively based on heat pipes and dynamic thermal metamaterials.

For a general TGP, the effectively extreme-large conductivity and rapidly passive heat spreading are obtained through cyclic two-phase fluid motions pumping by the continual condensation and evaporation of the working fluid[6,7]. Hence, the wicking structure for vapor chambers[6,7] (or a serpentine-arranged tube for oscillating heat pipes[6]) is indispensable to the motions of working fluid, while additional heat sink for generating condensate and thermal source for generating vapor are also required to assist the entire cyclic phase changes. Due to the spreading effect, TGP should possess high effective conductivity and exhibit a uniform thermal profile without temperature gradients. However, the tunable range of conductivity is passive and quite dependent on the combined effects of designed parameters, including the external heat input, working fluid, filling ratio, and the actual structural design. Thus, it is hard to achieve the active and continuous control in the full range of conductive demands without changing the external heat inputs or practically environmental parameters.

Compared with TGP, the tunable analog thermal material is obtained by modulating a single fluid without phase changes inside a bilayer structure, while its functionality is not limited to the effectively extreme-large conductivity and uniform thermal profiles at extreme velocity. By adjusting the fluid at arbitrary velocities, a full range of effective conductivity from near-zero to near-infinity can be actively and continuously observed without changing any structural and environmental parameters. Meanwhile, the fluid continuity and mobility further contribute to the inhomogeneous distributions of the effective conductivities at a specific velocity, which paves the most essential conditions for functional thermal meta-devices with different demands of temperature gradients. Hence, various manipulative behaviors, including enhanced transparency, field contortion, field inversion, and sensitive cloaking can be also significantly realized and switched in-situ by simply adjusting the velocities.

In general, tunable analog thermal material possesses some unique properties, including the single-phase working fluid, minimalist structure, actively and robustly tunable conductivity, and various behaviors of field distributions. It is believed that these aspects would be complementary to conventional techniques, and further motivate the thermal managements with multifarious behaviors in-situ.

Supplementary References

[1] Xu, L., Yang, S. and Huang, J. Designing effective thermal conductivity of materials of core-shell structure: Theory and simulation. *Phys. Rev. E* **99**, 022107 (2019).

[2] Li, Y., Zhu, K. J., Peng, Y. G., Li, W., Yang, T., Xu, H. X., Chen, H., Zhu, X. F., Fan, S. and Qiu, C. W. Thermal meta-device in analogue of zero-index photonics. *Nat. Mater.* **18**, 48 (2019).

[3] Olver, F. W. J. and Maximon, L. C. in NIST Digital Library of Mathematical Functions Ch. 10 (Version 1.0.18, release date 27 March 2018); http://dlmf.nist.gov/10release1.0.18 of2018-03-27.

[4] Guenneau, S., Amra, C., Anisotropic conductivity rotates heat fluxes in transient regimes. *Opt. Express* 2013, 21(5): 6578-6583.

[5] Yang, T., Vemuri, K. P., and Bandaru, P. R., Experimental evidence for the bending of heat flux in a thermal metamaterial. *Appl. Phys. Lett.* **105**, 083908 (2014).

[6] Thompson, S. M., Ma, H. B. Recent advances in two-phase thermal ground planes. *Ann. Rev. Heat Trans.* **18**, 101-153 (2015).

[7] Sigurdson, M., Liu, Y. W., Bozorgi, P., Bothman, D., MacDonald, N., Meinhart, C. A large scale titanium thermal ground plane. *Int. J. Heat Mass Trans.* **62**, 178-183 (2013).
